# A *Drosophila* computational brain model reveals sensorimotor processing

**DOI:** 10.1038/s41586-024-07763-9

**Published:** 2024-10-02

**Authors:** Philip K. Shiu, Gabriella R. Sterne, Nico Spiller, Romain Franconville, Andrea Sandoval, Joie Zhou, Neha Simha, Chan Hyuk Kang, Seongbong Yu, Jinseop S. Kim, Sven Dorkenwald, Arie Matsliah, Philipp Schlegel, Szi-chieh Yu, Claire E. McKellar, Amy Sterling, Marta Costa, Katharina Eichler, Alexander Shakeel Bates, Nils Eckstein, Jan Funke, Gregory S. X. E. Jefferis, Mala Murthy, Salil S. Bidaye, Stefanie Hampel, Andrew M. Seeds, Kristin Scott

**Affiliations:** 1 Department of Molecular and Cell Biology and Helen Wills Neuroscience Institute, University of California, Berkeley, CA USA; 2 University of Rochester Medical Center, Department of Biomedical Genetics, New York, NY USA; 3 Max Planck Florida Institute for Neuroscience, Jupiter, FL USA; 4 HHMI Janelia Research Campus, Ashburn, VA USA; 5 Department of Biological Sciences, Sungkyunkwan University, Suwon, South Korea; 6 Princeton Neuroscience Institute, Princeton University, Princeton, NJ USA; 7 Computer Science Department, Princeton University, Princeton, NJ USA; 8 Department of Zoology, University of Cambridge, Cambridge, UK; 9 Neurobiology Division, MRC Laboratory of Molecular Biology, Cambridge, UK; 10 Centre for Neural Circuits and Behaviour, The University of Oxford, Oxford, UK; 11 Department of Neurobiology and Howard Hughes Medical Institute, Harvard Medical School, Boston, MA USA; 12 Institute of Neurobiology, University of Puerto Rico-Medical Sciences Campus, San Juan, Puerto Rico; 13 Present Address: Eon Systems, San Francisco, CA USA

**Keywords:** Network models, Decision

## Abstract

The recent assembly of the adult *Drosophila melanogaster* central brain connectome, containing more than 125,000 neurons and 50 million synaptic connections, provides a template for examining sensory processing throughout the brain^1,2^. Here we create a leaky integrate-and-fire computational model of the entire *Drosophila* brain, on the basis of neural connectivity and neurotransmitter identity^3^, to study circuit properties of feeding and grooming behaviours. We show that activation of sugar-sensing or water-sensing gustatory neurons in the computational model accurately predicts neurons that respond to tastes and are required for feeding initiation^4^. In addition, using the model to activate neurons in the feeding region of the *Drosophila* brain predicts those that elicit motor neuron firing^5^—a testable hypothesis that we validate by optogenetic activation and behavioural studies. Activating different classes of gustatory neurons in the model makes accurate predictions of how several taste modalities interact, providing circuit-level insight into aversive and appetitive taste processing. Additionally, we applied this model to mechanosensory circuits and found that computational activation of mechanosensory neurons predicts activation of a small set of neurons comprising the antennal grooming circuit, and accurately describes the circuit response upon activation of different mechanosensory subtypes^6–10^. Our results demonstrate that modelling brain circuits using only synapse-level connectivity and predicted neurotransmitter identity generates experimentally testable hypotheses and can describe complete sensorimotor transformations.

## Abstract (web summary)

We create a computational model of the adult *Drosophila* brain that accurately describes circuit responses upon activation of different gustatory and mechanosensory subtypes and generates experimentally testable hypotheses to describe complete sensorimotor transformations.

## Main

The *Drosophila melanogaster* central brain connectome, comprising more than 125,000 neurons and 50 million synaptic connections, allows brainwide analyses of how the fly processes sensory information^1,2,11–16^. Although the *Drosophila* brain is comparatively small, a single *Drosophila* neuron may be connected synaptically to hundreds of downstream neurons, and interpreting how this connectivity relates to behaviour is not straightforward.

**Fig. 1:**
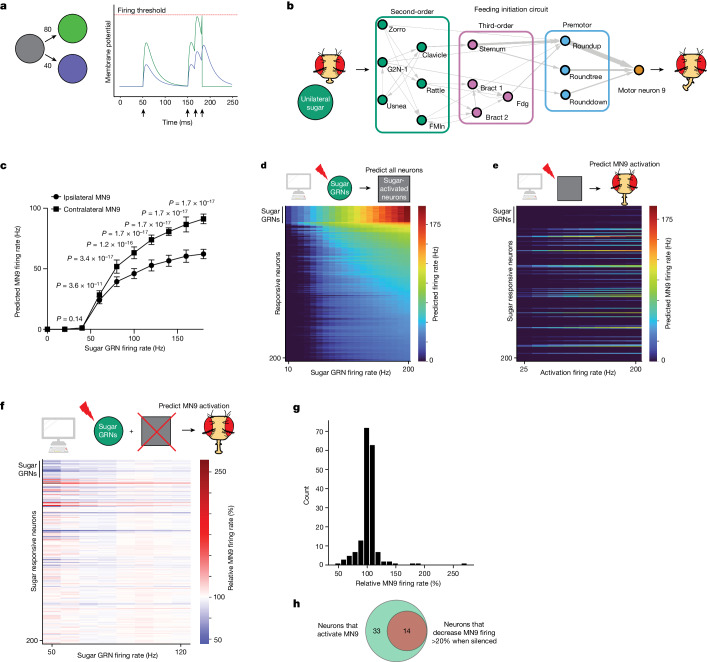
The computational model accurately predicts neurons that respond to sugar stimulation and neurons required for proboscis extension to sugar. **a**, Computational model schematic. Activation of the grey neuron at the times indicated by the arrows causes depolarization of the green and purple neurons in proportion to their connectivity from the grey neuron. Upon reaching the firing threshold, a neuron spikes, and its membrane potential is reset. **b**, Schematic of the proboscis extension response: unilateral sugar presentation causes proboscis extension towards the side of the fly with sugar. Arrow widths are proportional to the number of synapses connecting each set of neurons. **c**, Predicted MN9 firing rate of either the ipsilateral or contralateral MN9 in response to unilateral left hemisphere sugar GRN activation. Error bars represent s.d., Mann–Whitney *U* test. **d**, Heatmap depicting the predicted firing rates in response to unilateral 10–200 Hz sugar GRN firing. The *y* axis is ordered by firing rate at 200 Hz sugar activation, and depicts the top 200 most active neurons. **e**, Heatmap depicting the predicted MN9 firing rate when the top 200 responsive neurons are activated at 25–200 Hz; in **d** and **e**, the grey squares represent neurons that respond to sugar. **f**, Heatmap depicting the change in the contralateral MN9 firing rate in response to activation of sugar GRNs, while individually silencing each of the top 200 responsive neurons. The MN9 firing rate for each silenced neuron is normalized relative to the firing of MN9 when no neurons are silenced for each GRN activation frequency; In **e** and **f**, the *y* axis is ordered as in **d**. **g**, Histogram of the non-GRNs in **f** at 50 Hz. **h**, Venn diagram depicting the intersection between neurons predicted to activate MN9 and neurons predicted to cause a 20% decrease in MN9 firing when silenced.

To model the neural circuit mechanisms that generate behaviour, we implement a simple leaky integrate-and-fire model using the connection weights derived from the entire adult *Drosophila* central brain connectome of reconstructed electron microscopy neurons^1,17–20^, as well as neurotransmitter predictions for each neuron^3,21^. In this model, spiking of a neuron alters the membrane potential of downstream neurons in proportion to the connectivity from the upstream neuron^17^ (Fig. 1a and Methods); if a downstream neuron’s membrane potential reaches the firing threshold, that neuron, in turn, fires.

We implemented this model in the spiking neural network simulator Brian2 (ref. ^22^) using all Flywire neurons. The baseline firing of each neuron in our model is 0 Hz. All biophysical parameters are taken from previous *D.* *melanogaster* modelling or electrophysiology efforts^18,19^, from the synaptic weights from the Flywire connectome^1,21^ and from the neurotransmitter predictions^3^, except for *W*_syn_—the single free parameter of the model, which corresponds to the magnitude of the change in downstream membrane potential that results from a single excitatory or inhibitory synapse (Methods). By driving activity in a sparse set of neurons, the model predicts changes in downstream firing.

We examined the model’s ability to predict circuit activity in two systems: feeding initiation and antennal grooming. We began by examining the *Drosophila* feeding initiation circuit because it has well-defined taste sensory inputs and motor outputs that are contained in the Flywire electron microscopy volume. Thus, computational modelling of the feeding initiation circuit permits analysis of an entire sensorimotor circuit, in contrast to other sensorimotor circuits that require descending neurons, which are incomplete in the Flywire volume. Furthermore, gustatory sensory neurons that respond to sugar, water and bitter tastes have been identified in the electron microscopy volume^23^, permitting a detailed analysis of how these modalities interact. In addition, extensive experimental analysis provides a ground truth for computational studies^4,5,23–32^. We further assessed the performance of the model in another well-defined but non-overlapping circuit—the antennal grooming circuit—as an independent evaluation of the model^6–10^. As with the feeding initiation circuit, the antennal grooming circuit has well-defined sensory inputs, and a discrete, easily quantified behavioural output: antennal grooming behaviour. In both circuits, we tested specific predictions that the computational model generated using cell-type-specific genetic tools, optogenetics and functional imaging. We find that the model makes predictions consistent with our empirical observations, such as identification of neurons required for behavioural output. Thus, our computational model reduces the vast complexity of the connectome into simple, intuitive circuits.

**Extended Data Fig. 1:**
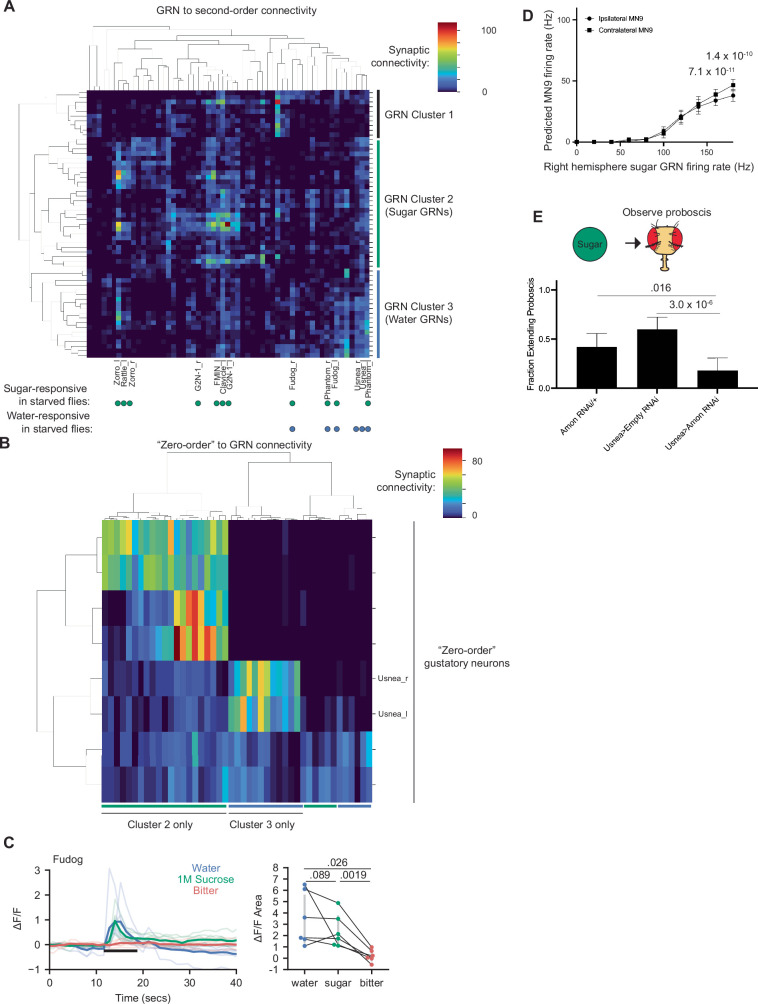
Identification of water and sugar GRN modality. A. Hierarchical clustering of GRNs and second-order gustatory neurons based on cosine similarity of connectivity of GRNs (y-axis) onto second-order neurons (x-axis). Below, the taste response properties of tested second-order neurons in starved flies is plotted (Shiu, Sterne et al.^4^), demonstrating that GRN cluster 2 synapses most strongly onto neurons that respond only to sugar in starved flies. B. Hierarchical clustering of “zero-order” gustatory neurons onto cluster 2 and 3 GRNs based on cosine similiarity. Black, green and blue lines designate which cluster from (A) each GRN in B belongs to. C. Calcium responses of the second-order neuron Fudog to stimulation of the proboscis in food-deprived flies. Quade’s test with Quade’s All Pairs test, using Holm’s correction to adjust for multiple comparisons. D. Predicted MN9 firing rate of either the ipsilateral or contralateral MN9 in response to unilateral right-hemisphere sugar GRN activation. Mean +/− Standard deviation, Mann-Whitney U-test. E. The fraction of flies exhibiting proboscis extension response upon 50 mM sucrose stimulation. Mean +/− 95% confidence intervals using Wilson’s score interval, Fisher’s exact test. n = 50 flies per genotype.

In *Drosophila* feeding initiation, detection of appetitive substances in hungry flies results in proboscis extension and consumption^33^. Gustatory receptor neurons (GRNs) on the body surface of the fly, including the labellum (tip of the proboscis) or the legs, respond directly to tastants and project to the primary taste centre of the insect brain—the suboesophageal zone (SEZ)^23,33–37^. GRNs respond to specific taste categories, such as appetitive sugar or aversive bitter compounds, resulting in acceptance (that is, proboscis extension and feeding) or avoidance, respectively^24,33,35,38^.To examine the neural circuits that influence feeding in response to taste detection, we focussed on four GRN categories: sugar, water, bitter and a fourth GRN category labelled by the ionotropic receptor Ir94e. Ir94e neurons respond to salt and the presentation of male genitals, but the exact tastants Ir94e neurons respond to are not well understood^31,33,37,38^. These GRNs have been identified and classified previously in the electron microscopy brain volume^23^; we verify and expand on this classification by clustering on the basis of connectivity and comparing this clustering with response properties of second-order neurons (Extended Data Fig. 1a–c and Methods).

When a fly encounters sugar, activation of appetitive GRNs results in activation of proboscis motor neurons (MNs) (Fig. 1b). The proboscis consists of three segments: the rostrum, the haustellum and the lip-like labella, controlled by the activity of 16 MNs^32^. We find that computational activation of labellar sugar-sensing GRNs activates several proboscis MNs involved in feeding, including MNs 6, 8, 9 and 11 (Fig. 1c, Extended Data Fig. 1d and Supplementary Table 1)^39^. Consistent with the model’s predictions, MN9 and MN11 have been shown previously to respond to sugar stimulation in vivo^5,39^. In total, we find that the computational model can model a complete sensorimotor transformation.

To assess the ability of our computational model to predict the composition and function of the feeding initiation circuit, we focussed specifically on the activity of MN9, which controls rostrum lifting during proboscis extension^5,32^. The rostrum is the largest portion of the proboscis, permitting quantification of MN9 activity by measuring rostrum lifting. Although the exact correlation between MN9 firing rate and rostrum lifting is not known, we assume that increased MN9 firing rates correspond with increased rostrum lifting probability. Remarkably, unilateral sugar GRN activation activates the contralateral MN9 more strongly compared with the ipsilateral MN9 when either the left (Fig. 1c) or the right (Extended Data Fig. 1d) hemisphere GRNs are activated, consistent with behavioural experiments showing that unilateral taste detection on the legs promotes proboscis extension that is curved and directed towards the food source^26,40^. Thus, we show that in silico sensory activation produces MN activity that is consistent with the observed behaviour of the fly taste sensorimotor circuit.

To confirm that our computational activation of MN9 depends on the actual connectivity weights determined from the fly connectome, we tested whether distorting synaptic weights would impair the ability of sugar sensory neurons to activate MN9. In these experiments, connectivity weights were shuffled randomly (while maintaining the global connectivity weight distribution). Although modelling using the correct connectome results in robust activation of MN9 in 100% of simulations when sugar-sensing neurons are activated at 100 Hz, only 1 of 100 shuffled simulations did (Supplementary Table 1d). Therefore, the predictive accuracy of our computational model depends on the actual connectivity weights of the fly connectome.

We next examined whether the computational model could accurately predict the neuronal cell types that are known to compose the feeding initiation circuit^4^. We first examined the neural network activated upon unilateral sugar GRN activation. We note that, given the variety of assumptions the model relies upon, absolute firing rate predictions are unlikely to be accurate; therefore, we examined network activity upon sugar GRN activation ranging from 10 to 200 Hz (Fig. 1d). We find that increasing sugar GRN firing rate increases activity of MN9, as well as MNs 6, 8 and 11. Of the 127,400 neurons modelled, we found that 45 are predicted to respond to 10 Hz sugar GRN activation, and 455 to 200 Hz (Supplementary Table 1). Activated neurons are defined as neurons that have greater than 0 Hz firing. Thus, the computational model predicts a large network activated by sugar taste detection that includes known sugar-responsive MNs.

Sugar taste detection influences activity in nutritive state and memory circuits, and modulates a broad range of behaviours, including feeding, oviposition and foraging^33,37,41^. To specifically evaluate the subset of predicted sugar-responsive neurons that influence feeding initiation, we performed two further in silico experiments. First, as a strategy to identify neurons that drive feeding initiation, we computationally stimulated each of the top sugar-responding neurons in the network to identify those that drive activity in MN9 (Fig. 1e). Second, to identify neurons required for feeding initiation to sugars, we computationally activated sugar GRNs, silenced each of the top 200 sugar-responsive neurons one at a time, and measured the change in predicted MN9 firing (Fig. 1f,g). For these silencing experiments, we activated sugar-sensing neurons at frequencies ranging from 50 to 120 Hz in 10 Hz increments. Neurons that our model predicts to be required for feeding initiation will have decreased MN9 firing when silenced. We defined neurons predicted to cause a silencing phenotype as any neuron whose silencing causes MN9 firing to be 80% or lower compared with control MN9 firing at any of the eight sugar activation frequencies tested (50, 60, 70, …120 Hz; Fig. 1f). In general, silencing of individual neurons had the greatest effect when sugar GRNs were activated at low frequencies, implying greater redundancy in the circuit as sensory stimulation increases. In total, our analyses identified 47 neurons predicted to be sugar-responsive, and sufficient for feeding initiation. Of these 47 neurons, 14 are also predicted to be required for MN9 activity (Fig. 1h).

**Extended Data Fig. 2:**
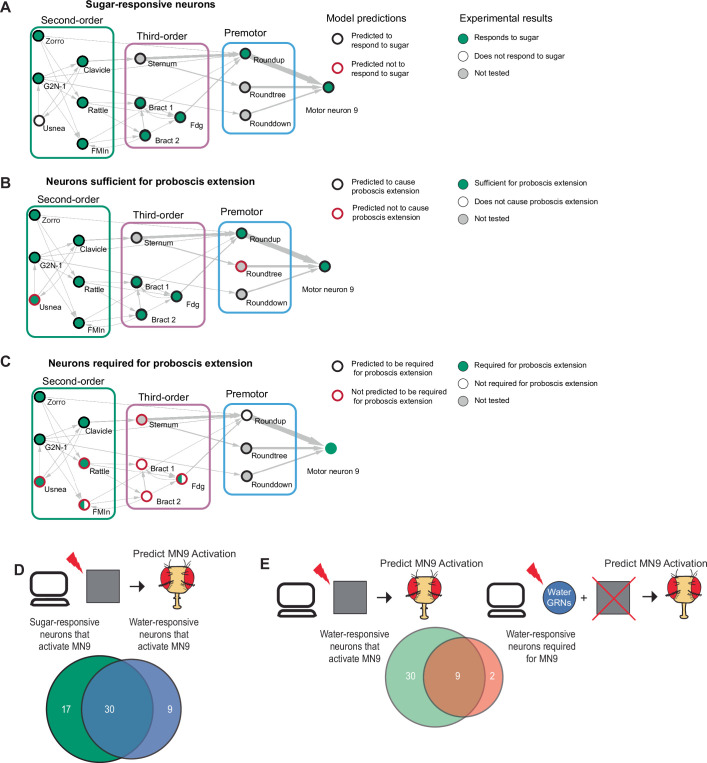
A comparison between predicted water, sugar, bitter and Ir94e activation. A-C. Schematics showing which cell types either respond to sugar (A), are sufficient for proboscis extension (B), or are required for proboscis extension to sugar (C). The color of the circle perimeter corresponds to the model predictions, and the inside of the circle represents experimental results. There are mixed results for whether Fdg or FMIn are required for proboscis extension to sugar. D. Venn diagram of the sugar-responsive neurons that are sufficient for MN9 activation and water-responsive neurons sufficient for MN9 activation. E. Venn diagram comparing predicted water-responsive neurons sufficient for MN9 and water-responsive neurons required (i.e., predicted to reduce MN9 firing >20%) for MN9 activity. GRNs are excluded from this analysis. (A-C): Figure adapted with permission from Shiu, Sterne et al.^4^; CC-BY License.

We next evaluated whether the predicted neurons for feeding initiation include neurons shown experimentally to participate in feeding initiation behaviour. Previous experimental studies identified ten neural classes that respond to sugar, and are sufficient for proboscis extension^4^ (Fig. 1b and Extended Data Fig. 2a–c). Our computational model correctly predicts that all ten cell types respond to sugar (Supplementary Table 2). Of these ten neurons, eight are predicted correctly to be sufficient to activate MN9 (ref. ^4^) (Supplementary Table 2). We previously found that five of the ten are required for sugar feeding initiation^4^ (Supplementary Table 2). Of these five, three are predicted by our computational model to cause a greater than 20% decrease in MN9 firing, and one of the others is predicted to cause a statistically significant decrease in MN9 firing, but less than 20%, when silenced. Although the model predictions generally match previous experimental results, there are some deviations. For example, the model fails to correctly predict that the Phantom cell type will activate MN9 (ref. ^4^) (Fig. 1e, Extended Data Fig. 2c and Supplementary Table 2). This cell type is predicted to be inhibitory. Phantom strongly synapses onto Scapula—a neuron that is also predicted to be inhibitory; Scapula, in turn, synapses onto Roundup, the pre-MN with the strongest predicted silencing phenotype. We speculate that activation of Phantom inhibits Scapula, potentially permitting Roundup and MN9 firing. Because the basal firing rate of all neurons in the model is 0, activation of inhibitory neurons in the model, in the absence of other input, cannot alter the firing of downstream neurons. A further explanation for incorrect predictions could be neuromodulation, which is not accounted for in our model. Particular neurons may be subject to neuromodulation, causing their activity to be different from predictions on the basis of connectivity. Alternatively, neurons that express neuromodulators may be poorly modelled. We speculated that the Usnea cell type, which has a strong experimental activation and silencing phenotype^4^ yet is not predicted to be either necessary or sufficient for proboscis extension, might be neuropeptidergic. To test this, we performed cell-specific knockdown of the gene *Amontillado*—a prohormone convertase required for neuropeptide processing in *Drosophila*^42,43^. Knockdown of *Amontillado* phenocopied the Usnea silencing phenotype (Extended Data Fig. 1e), indicating that Usnea activity may require neuropeptide processing. Additionally, incorrect neurotransmitter predictions or other assumptions of the model may explain discrepancies between the prediction of our model and our experimental results. Despite these limitations, overall, this analysis demonstrates that our computational model correctly identifies known neurons in a sensorimotor circuit.

**Fig. 2:**
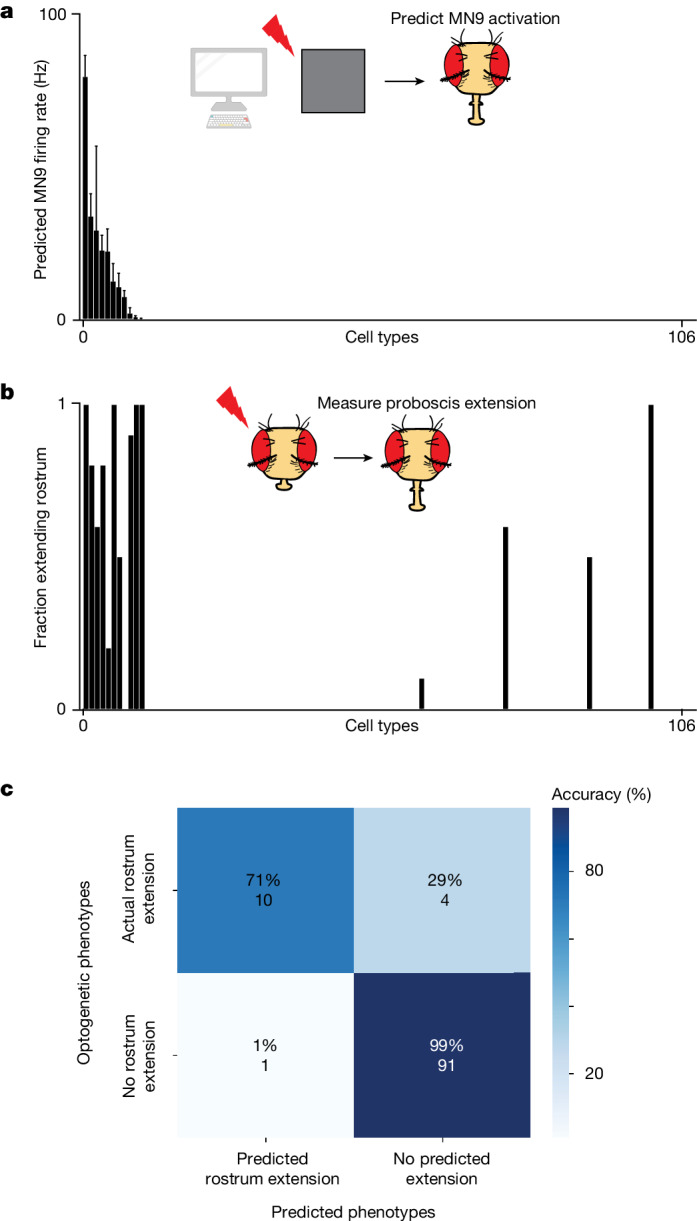
The computational model predicts neurons that cause proboscis extension. **a**, Predicted MN9 firing rates when each of 106 cell types are activated computationally at 50 Hz. Cell types are ordered by predicted MN9 firing rate. **b**, Fraction of flies extending the rostrum—the segment of the proboscis controlled by MN9—in response to optogenetic activation; cell types are ordered as in **a**. Where several split-GAL4 lines for a cell type were tested, the line with the highest extension rate is plotted; *n* = 10 flies per cell type. **c**, Confusion matrix showing the accuracy of MN9 activation predictions and the number of cell types in each category. The rostrum was predicted to extend if MN9 was predicted to have non-zero firing as a result of 50 Hz cell-type activation.

As an independent assessment of whether the computational model accurately predicts neurons that elicit MN9 activity, the output of our sensorimotor circuit, we compared optogenetic activation phenotypes with their corresponding computational activation phenotypes. To do this in a non-biased way, we performed a screen in which we optogenetically activated individual neuronal cell types with split-GAL4 lines and monitored the activity of MN9. The SEZ split-GAL4 collection labels 138 cell types in the SEZ—the primary feeding region of the brain^44^. We identified 106 of these labelled cell types in the Flywire volume. Next, we crossed these split-GAL4 lines to create flies expressing the light-gated cation channel CsChrimson. We then optogenetically activated these neurons, and measured whether MN9 is activated by observing rostrum extension. We compared the predicted in silico MN9 activation phenotypes of these cell types when we activate them between 10 Hz and 200 Hz with the actual optogenetic activation MN9 phenotypes we observed. When we activate each cell type at 50 Hz, 11 are predicted to activate MN9 (that is, elicit MN9 firing greater than 0 Hz); notably, 10 of 11 of these cell types actually do elicit rostrum extension when optogenetically activated (Fig. 2a–c and Supplementary Table 3). Furthermore, of the 95 predicted not to elicit proboscis extension due to 50 Hz activation, just 4 have non-zero rostrum extension. Activation of these cell types at 200 Hz, rather than 50 Hz, results in the addition of five false positives. At 10 Hz activation, six cell types are predicted to cause MN9 activation; of these five, six do indeed cause proboscis extension. Thus, the computational model can predict the activation phenotypes of a non-biased sample of cell types at greater than 90% accuracy.

**Fig. 3:**
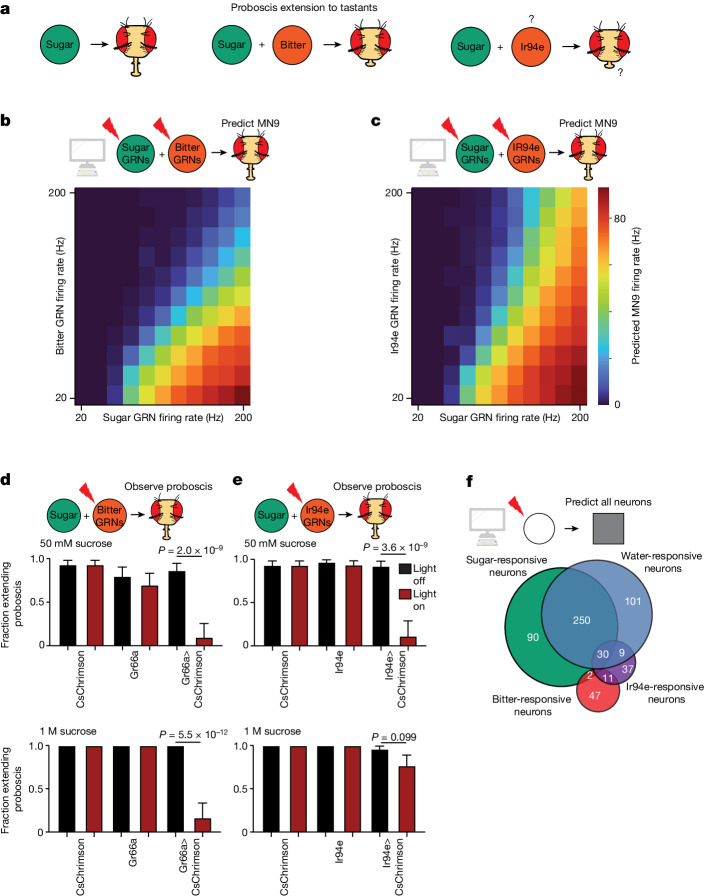
The computational model correctly predicts that Ir94e neurons are aversive but fail to inhibit proboscis extension to a strong sugar stimulus. **a**, Schematic outlining the previously known and unknown roles of sugar, bitter and Ir94e neurons. Question marks indicate that the exact substrate(s) that activate Ir94e neurons are not known, nor is it known whether Ir94e activation influences proboscis extension. **b**,**c**, Heatmap depicting the predicted MN9 firing rates in response to the combination of sugar GRN firing and bitter (**b**) or Ir94e (**c**) GRN activation. **d**,**e**, Fraction of flies exhibiting PER upon 50 mM sucrose stimulation or 1 M sucrose stimulation when Gr66a/bitter GRNs (**d**) or Ir94e GRNs (**e**) are optogenetically activated. Red bars indicate red light condition; *n* = 26–32, see Supplementary Table 9 for exact values. Mean ± 95% confidence intervals using Wilson’s score interval, Fisher’s exact test. **f**, Venn diagram showing the number and overlap of neurons that respond to sugar GRN (green) or water GRN (blue) activation that elicits 40 Hz MN9 firing, as well as bitter GRN (red) or Ir94e GRN activation (purple) activated to reduce 40 Hz MN9 firing to 1 Hz.

The accuracy of the model indicates that it provides a powerful platform to discover how different taste modalities are processed to influence feeding initiation. We first tested whether the model can predict the response to coactivation of both an attractive sugar stimulus and an aversive bitter stimulus. Bitter detection inhibits proboscis extension motor activity^45^ (Fig. 3a). Indeed, the addition of bitter GRN activity to sugar GRN activation in our model resulted in an inhibition of MN6 and MN9 (Fig. 3b and Supplementary Table 4). We previously found, using calcium imaging, that bitter GRN activation inhibits the sugar pathway at the level of pre-MNs^4^, consistent with the predictions of the computational model (Supplementary Table 4).

We next examined the predicted circuit activity caused by GRNs labelled by the ionotropic receptor Ir94e; these neurons have been identified previously in the electron microscopy volume^23^. Ir94e neurons respond to low salt concentrations and the presentation of male genitals, among other substances^31,38^, and are suggested to play a role in mediating attraction to low salt^31^. However, the role they play in proboscis extension has not been described. Notably, the computational model predicted that activation of Ir94e GRNs, rather than promoting MN9 firing, inhibits MN9 firing (Fig. 3c). Therefore, we tested whether optogenetic activation of Ir94e GRNs is sufficient to inhibit proboscis extension, similar to bitter activation. Indeed, we found that optogenetic activation of Ir94e GRNs or bitter GRNs was sufficient to inhibit the proboscis extension to 50 mM sucrose, as our modelling predicted (Fig. 3d,e). Interestingly, we noted a quantitative difference between the model’s predictions for bitter versus Ir94e activation. Strong bitter activation is predicted to eliminate MN9 firing to strong sugar stimulation, but strong activation of Ir94e neurons is not predicted to do so (Fig. 3b,c). We therefore tested the proboscis extension response (PER) to 1 M sucrose while optogenetically activating bitter or Ir94e GRNs. Optogenetic bitter activation eliminated consumption of 1 M sucrose (Fig. 3d), but Ir94e activation did not (Fig. 3e). Thus, we conclude that Ir94e GRN activity inhibits proboscis extension, but fails to fully inhibit proboscis extension to strong sugar stimuli. These results indicate that our computational model can predict previously unknown circuit functions and properties.

**Fig. 4:**
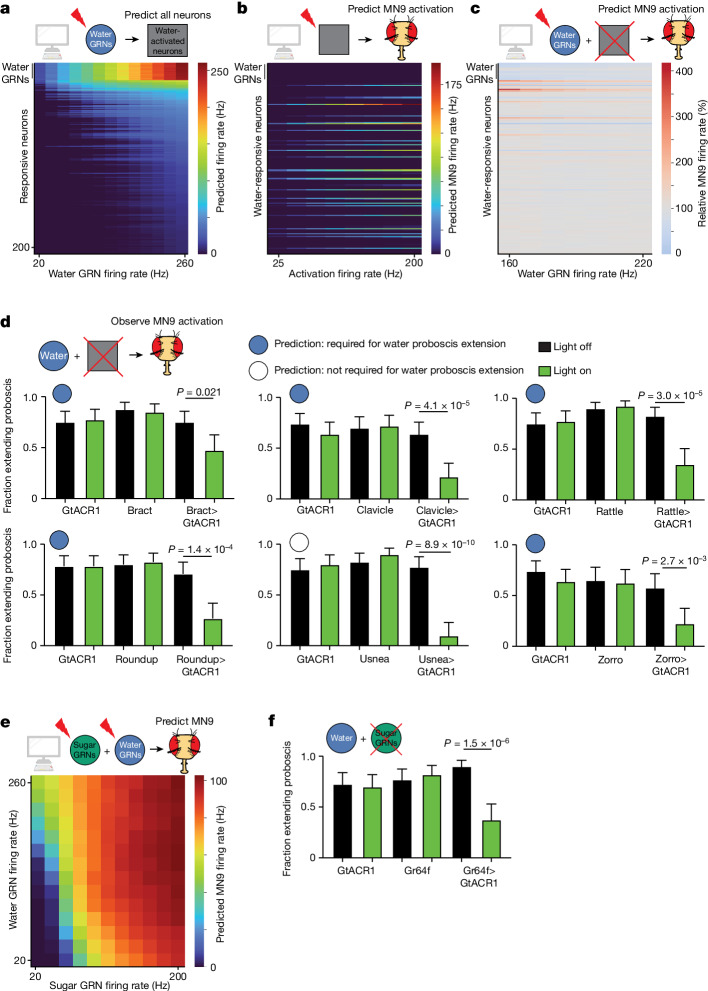
The computational model correctly predicts that the sugar and water pathways share components and additively promote proboscis extension. **a**, Heatmap depicting the predicting firing rates in response to 20 to 260 Hz water GRN firing. The *y* axis is ordered by firing rate at 260 Hz water activation. **b**, Heatmap depicting the predicted MN9 firing rate when the top 200 responsive neurons are activated at 25–200 Hz. **c**, Heatmap depicting the change in MN9 firing rate in response to activation of water GRNs at the specified firing rate, while individually silencing each of the top 200 responsive neurons. **d**, The fraction of flies exhibiting PER upon water stimulation. Green bars indicate green light condition; *n* = 30–50; see Supplementary Table 9 for exact values. Open and filled circles represent whether the computational model predicted a greater than 20% decrease in MN9 firing at 160 Hz water GRN stimulation. **e**, Heatmap depicting the predicted MN9 firing rates in response to the combination of sugar and water GRN activity. **f**, The fraction of flies exhibiting PER upon water stimulation. *n* = 39–40. **d**,**f**, Mean ± 95% confidence intervals, Fisher’s exact test.

Finally, we sought to predict how water taste detection influences feeding initiation. The degree to which sugar GRNs and water GRNs activate pathways that are distinct or shared is unknown. We found that activation of water GRNs in our model activates many downstream neurons that are also activated by sugar stimulation. (Fig. 3f, Fig. 4a and Supplementary Tables 1 and 6). In particular, comparing neurons activated by sugar GRNs with those activated by water GRNs, at a stimulation frequency at which each pathway activates MN9 at 40 Hz, predicted that the sugar pathway activates 377 neurons, while the water pathway activates 391 neurons. Of these, more than half (250), are shared between the two circuits (Fig. 3f and Supplementary Table 4). We also examined bitter responsive neurons and Ir94e responsive neurons at the minimal activation sufficient to reduce 40 Hz MN9 firing to 1 Hz. Only two neurons were common between sugar and bitter activation, and 30 between sugar and Ir94e activation, demonstrating segregation of neurons activated by aversive and appetitive taste (Fig. 3f). This prediction is consistent with our previous calcium imaging experiments demonstrating that, across nine sugar-responsive cell types, zero respond to a mixture of bitter compounds^4^. In contrast, our model predicts central neurons that respond to both sugar and water taste activation, as well as sugar-specific and water-specific neurons, consistent with brainwide calcium imaging studies^29,46^.

To identify interneurons that compose the water feeding initiation circuit, we used the computational model to analyze the water-responsive neurons that influence MN9 activity. We stimulated the top 200 neurons that are predicted to respond to water, and identified the subset that computationally activates MN9 (Fig. 4b). Next, we also computationally activated water-sensing GRNs, silenced each water-responsive neuron, and monitored the change in MN9 activity (Fig. 4c and Extended Data Fig. 2d). Using our computational model, we identified 39 water-responsive neurons that are also sufficient for MN9 activation. Of these 39, 30 are also predicted to be activated by sugar GRNs (Extended Data Fig. 2d). Furthermore, we identify nine neurons predicted to be both necessary and sufficient for water feeding initiation (Extended Data Fig. 2e). As with sugar, we defined a neuron predicted to be required for water feeding initiation as any neuron that, when silenced, caused MN9 firing to be less than 80% of that of the unsilenced control.

**Extended Data Fig. 3:**
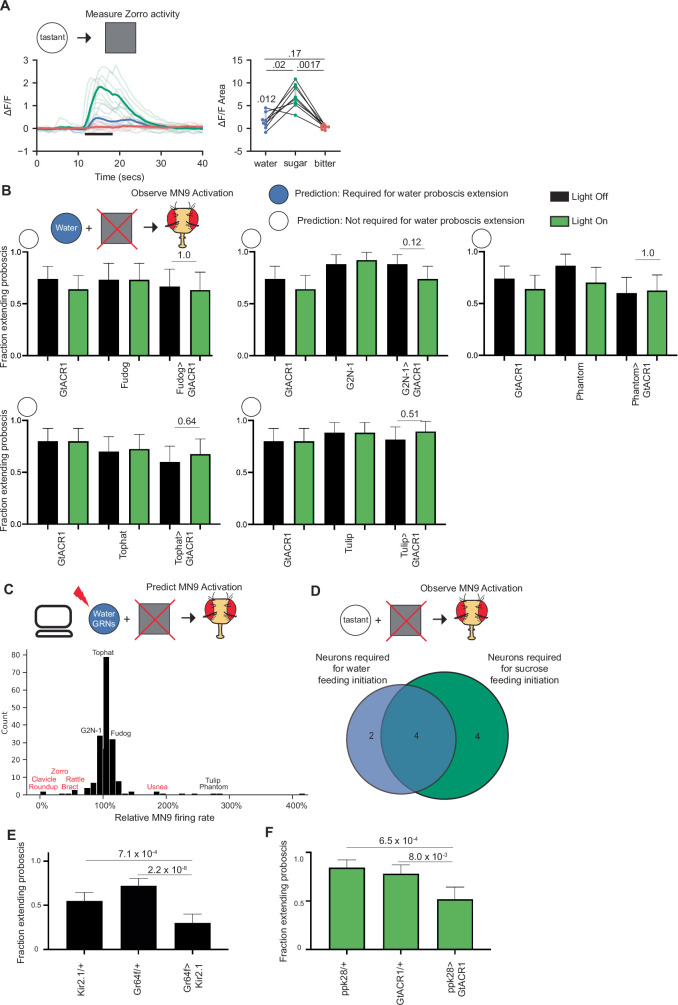
A comparison between predicted water silencing phenotypes and optogenetic silencing. A. Calcium responses of the second-order neuron Zorro to stimulation of the proboscis in pseudodessicated flies. Quade’s test with Quade’s All Pairs test, using Holm’s correction to adjust for multiple comparisons (testing between tastants), or Wilcoxon one-sided test (testing if different than 0). B. The fraction of flies exhibiting proboscis extension response upon water stimulation. Green bars indicate green light condition. n = 40–50. ns: not significant. Open and filled circled represent whether the computational model predictions a 20% decrease in MN9 firing at 160 Hz water GRN stimulation. C. Histogram of water silencing predictions of the non-GRNs in Fig. 4c at 160 Hz, with tested cell types labelled. Y-axis depicts the number of neurons in each bin. Cell types in red have an experimental water silencing phenotype. D. Comparison of neurons found to be required for feeding initiation to either water or 50 mM sucrose (Shiu, Sterne et al.^4^), when silenced with the anion channelrhodopsin GtACR1. E. The fraction of flies exhibiting proboscis extension response upon water stimulation, n = 90–100. F. The fraction of flies exhibiting proboscis extension response upon 50 mM sucrose stimulation. n = 45–56. B, E, F: Mean +/− 95% confidence intervals (Wilson Interval Score), Fisher’s exact test.

To test these predictions experimentally, we performed calcium imaging on two neurons predicted to respond to water: Fudog and Zorro. We found that both neurons indeed responded to water (Extended Data Figs. 1c and  3a). Additionally, we examined six neurons predicted to have water silencing phenotypes. Five of these, when silenced optogenetically, indeed decreased significantly proboscis extension to water, while a sixth, G2N-1, did not (Fig. 4d and Extended Data Fig. 3b,c). We also examined five neurons that respond to computational water activation, but are not predicted to cause a water silencing phenotype. Of these five neurons, four did not have a water silencing phenotype, as predicted, although one, Usnea, did decrease proboscis extension significantly when silenced with GtACR1 (Fig. 4d and Extended Data Fig. 3b,c).

Our computational model predicts that the water and sugar pathways share a common set of neurons (Extended Data Fig. 2d,e). Do these shared neurons contribute to feeding initiation? Our calcium imaging experiments (Extended Data Figs. 1c and 3a) combined with previous experiments^4^ confirm that five neurons predicted to respond to sugar and water do respond to both sugar and water in vivo: Clavicle, Fudog, Phantom, Rattle and Zorro. Moreover, four of these neurons had been shown previously to be sufficient for proboscis extension, and three are also required for sugar feeding initiation^4^. All three are among the neurons we found experimentally to be required for feeding initiation to water, as predicted (Fig. 4d). Furthermore, the two other cell types we found experimentally to be required for water—Bract and Roundup—are also predicted to respond to both water and sugar (Supplementary Tables 1 and 6), and have been found to respond to sugar^4^. However, previous calcium imaging studies did not identify water responses in these two cell types^4^. This discrepancy may reflect the greater sensitivity of the behavioural silencing experiments compared with calcium imaging of water responses^4^. Finally, a further cell type, Usnea, has been shown to respond to water, but not sugar^4^; our model correctly predicts Usnea responds to water, but incorrectly predicts that it will also respond to sugar. Usnea has previously been shown to be required for feeding initiation to sugar, and our *Amontillado* RNAi experiments indicate that it may be neuropeptidergic. We find that it is also required for proboscis extension to water (Fig. 4d). Usnea synapses directly onto both sugar and water GRNs (Extended Data Fig. 1b), and may tune the response of these neurons. Thus, we identify a set of neurons involved in the processing of both sugar and water.

To explore the relationship between the water and sugar pathways, we computationally activated both sugar and water GRNs simultaneously and examined the effect on MN9. Our computational modelling predicts that activation of water and sugar GRNs work synergistically to promote MN9 firing (Fig. 4e and Extended Data Fig. 3d). If sugar and water do act synergistically, then both sugar GRNs and water GRNs may be involved in water consumption. Only water GRNs have been implicated in proboscis extension to water; we asked whether sugar GRNs might also be required. Indeed, silencing of sugar GRNs reduced the fraction of flies that extended their proboscis to water (Fig. 4f and Extended Data Fig. 3e). Further, silencing water-sensing neurons reduced consumption of 50 mM sucrose, although a confound is that these water-sensing neurons are known to respond to this concentration of sucrose (Extended Data Fig. 3f). In total, our computational modelling, optogenetic behaviour experiments and functional imaging indicate that the water and sugar pathways share, at least in part, common components to form an appetitive consumption pathway.

**Fig. 5:**
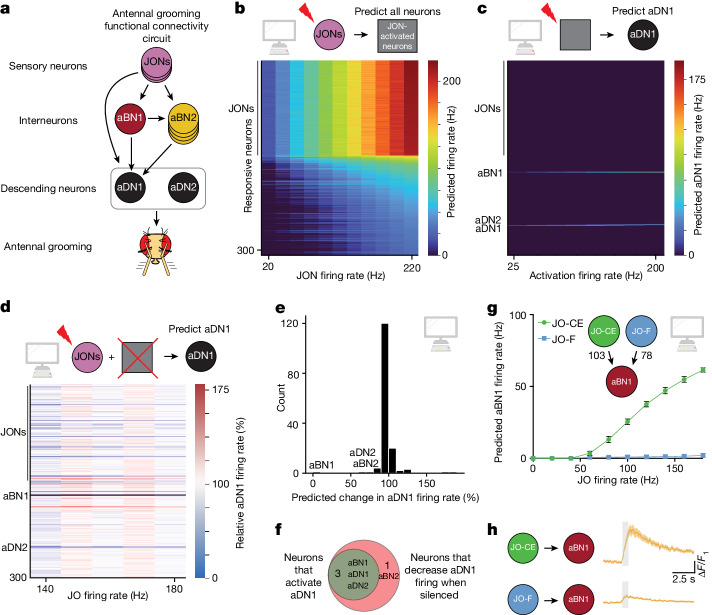
The computational model correctly identifies key neurons in the antennal grooming circuit as well as subtype circuit responses. **a**, Schematic of the antennal grooming circuit. Arrows represent known functional connectivity^8^. Grey oval around aDNs indicates that JONs activate aDNs, but exactly which aDNs are not known. **b**, Heatmap depicting the predicting firing rates in response to 20–220 Hz JON firing; 147 JONs were activated, and are the neurons that have the highest firing rates. Neurons are ordered by firing rate at 220 Hz. **c**, Heatmap depicting the predicted aDN1 firing rate when the top 300 responsive neurons are activated at 25–200 Hz. **d**, Heatmap depicting the change in aDN1 firing rate in response to activation of JOs at the specified firing rate, while individually silencing each of the top responsive neurons. **e**, Histogram of the predicted change in aDN1 firing rate as a result of silencing each non-JONs, when JONs are activated at 140 Hz. The *y* axis depicts the number of neurons in each bin. Neurons previously identified are labelled. **f**, Venn diagram depicting the overlap between neurons predicted to be sufficient to activate aDN1 at greater than 2 Hz and neurons required for aDN1 activation. **g**, JO subtype connectivity onto aBN1 and predicted aBN1 firing in response to JO activation at the specified rate. Error bars, s.d. **h**, Calcium imaging of aBN1 in response to optogenetic activation of each subtype. The Δ*F*/*F* average ± s.e.m. is shown; *n* ≥ 5 flies tested. Shaded bar indicates when a red light pulse was delivered.

To test the general applicability of the computational model to study sensorimotor processing, we sought to determine whether it could predict circuit properties in another system—the well-studied antennal grooming circuit^6–10^. In this system, activation of a set of mechanosensory neurons in the Johnston’s organ—a chordotonal organ in the antennae—elicits grooming of the antennae^8,47^ (Fig. 5a). These mechanosensory neurons, abbreviated JONs, synapse onto two interneuron types, named antennal grooming brain interneurons 1 and 2 (aBN1 and aBN2), which in turn synapse onto two descending neurons, aDN1 and aDN2 (ref. ^8^). There is a single aBN1 per hemisphere, while there are several aBN2 neurons per hemisphere. Each of these cell types—aBN1, aBN2, aDN1 and aDN2—are sufficient for antennal grooming, while aBN1 and aBN2 are each at least partially required for antennal grooming^8^.

**Extended Data Fig. 4:**
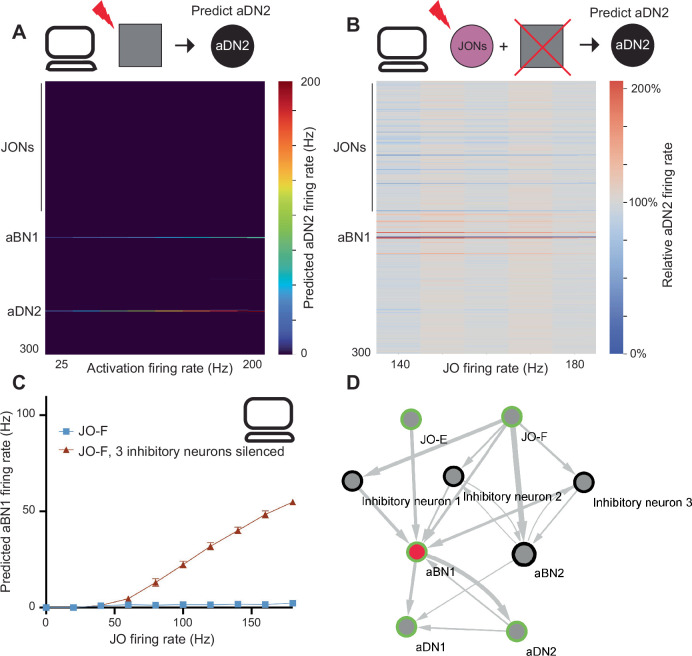
The computational model predicts neurons that elicit aDN2, and neurons that inhibit aBN1. A. Heatmap depicting the predicted aDN2 firing rate when the top 300 responsive neurons are activated at 25-200 Hz B. Heatmap depicting the change in aDN2 firing rate in response to activation of JOs at the specified firing rate, while individually silencing each of the top responsive neurons. C. Predicted aBN1 firing in response to JO-F activation at the specified rate. Red triangles, JO-F activation with co-silencing of three inhibitory neurons. Error bars represent one standard deviation. D. Schematic of the circuit, with neurons sufficient for antennal grooming in green, and required for antennal grooming in red. Width of arrows is proportional to number of synaptic connections.

We first sought to test whether the computational model could identify the previously described neurons in the circuit. We activated a set of 147 previously identified JONs of the JO-C, JO-E, JO-F and JO-m subclasses^8,47^. Indeed, the model identified that aBN1, aBN2, aDN1 and aDN2 respond to JON activation (Fig. 5b and Supplementary Table 5). To determine which of these JON-responsive neurons might drive antennal grooming, we computationally activated these neurons and asked whether they could elicit activity in either of the two descending neurons that evoke antennal grooming: aDN1 or aDN2 (Fig. 5c and Extended Data Fig. 4a). Next, we asked, among the top neurons predicted to respond to JON activation, which are required for activation of aDN1 or aDN2 (Fig. 5d and Extended Data Fig. 4b). Notably, only four neurons, beyond aDN1 itself, were identified that could elicit aDN1 activity: aBN1, aDN2 and two other neurons that elicited less than 2 Hz aDN1 activity (Fig. 5c and Supplementary Table 7). Moreover, only three neurons, besides aDN1 itself, were identified that reduced aDN1 activity by more than 20% at 140 Hz JON activation: aBN1; a descending member of the BN2 class; and aDN2 (Fig. 5d–f). Thus, the computational model identifies members of each of the previously identified critical nodes of the antennal grooming circuit purely from knowledge of the sensory inputs and descending outputs.

We next tested how different JON subpopulations influence antennal grooming. JONs send their projections to the antennal mechanosensory and motor center in the ventral brain. JO-C and JO-E neurons respond to antennal vibrations and project medially into the antennal mechanosensory and motor center, while JO-F neurons project into a distinct region^8^. Optogenetic activation of both JO-CE and JO-F neurons is sufficient to trigger antennal grooming, but it is not known whether these two populations generate distinct patterns of downstream firing. Both JO-CE and JO-F neurons synapse onto aBN1 (103 and 78 synapses, respectively; Fig. 5g), raising the possibility that they elicit grooming by activating aBN1.

Our computational model predicts that, whereas JO-CE neurons will elicit robust aBN1 activity, JO-F neurons will not, despite synapsing directly onto aBN1 (Fig. 5g). To test this prediction, we optogenetically activated each population of JONs and performed calcium imaging in aBN1. Consistent with the prediction of this model, JO-CE activated aBN1 robustly, but JO-F neurons did not (Fig. 5h). Why do JO-F neurons fail to activate aBN1 robustly? We identified three putative inhibitory neurons that are directly postsynaptic to JO-F neurons and synapse directly onto aBN1. Computational silencing of these three neurons permits JO-F neurons to activate aBN1, but this remains to be tested empirically (Extended Data Fig. 4c,d). Our analysis of the antennal grooming circuit demonstrates that our computational model can provide insights into complex circuits, purely from knowledge of sensory input and descending output. We demonstrate that modelling brain circuits purely from connectivity and neurotransmitter identity is sufficient to reliably describe, at least at a coarse level, entire sensorimotor transformations.

In conclusion, we report a computational model on the basis of connectivity and neurotransmitter predictions of the entire fly connectome that can predict circuit neural activity, the neurons required for activation of output neurons and the integration of several sensory modalities. We use the model to create predictions of the sugar, water, bitter and Ir94e pathways and validate many of these predictions experimentally. We show that the Ir94e neurons, previously considered to be attractive, instead inhibit proboscis extension. The results of our modelling indicate that sugar, bitter and Ir94e GRNs activate generally distinct populations of neurons. In contrast, sugar and water GRNs activate many of the same central neurons as well as sugar-specific and water-specific neurons. In addition, we recapitulate the antennal grooming circuit purely from sensory input and descending output, and identify a subpopulation of JONs that, despite strong connectivity onto aBN1, fail to activate it. These studies demonstrate the power of computational modelling to explain sensory processing features in complex networks.

Our analysis of the taste and antennal grooming circuits shows we can model local sensorimotor transformations in the taste and antennal grooming circuits. The computational model, implemented in the widely used Brian2 library^22^, allows for perturbations that are easily interpreted. We believe our computational model will be a useful tool for the study of sensorimotor transformations and the exploration of interactions between overlapping neural pathways (for example, sweet-bitter, sweet-water and so on).

## Connectome models

We implement here a brainwide leaky integrate-and-fire model—one of the simplest biologically plausible neural models. The recent creation of connectome datasets has also coincided with methods to model these connectomes. Two different approaches have been used thus far to analyze the recent *Drosophila* hemibrain and larval connectomes^13,16^. First, graph theoretical approaches, such as the probabilistic graph traversal model^13^, or the signal cascade approach^16^ can be used to model information flow in the *Drosophila* brain. These models estimate the path length from one neuron to another on the basis of probabilistic traversal through the connectome depending on connection strength^13,16^. However, because these models measure traversal length, rather than a measure of activation, they are unlikely to be useful for predicting what neurons contribute most to a given circuit. A second approach is to train a deep neural network to perform a task, and measure how neurons in that model compare with actual neurons in the brain^15,48^. However, these approaches generally use markedly more free parameters (for example, 734 in the case of ref. ^15^), and need a clearly defined behavioural task.

In contrast to these approaches, our computational model generates an intuitive readout, changes in spiking rates of neurons, with no training of the model necessary. The model permits hypotheses about the function of particular neurons, and allows for modelling of the interactions of circuits that were previously studied only in isolation. For example, by activating neurons involved in locomotion simultaneously with sugar-sensing neurons, which are known to inhibit locomotion, Sapkal et al. use our computational model to correctly identify neurons that regulate walking, thereby identifying circuits involved in foraging^49^.

What are the requirements for our model to make accurate predictions, and what circuits or conditions might result in poor modelling? Our model failed to predict behavioural results in the SEZ split-GAL4 screen (Fig. 2) when the neurons tested were predicted to be inhibitory (that is, Tentacular or Phantom) or neuromodulatory (Usnea). We conclude that circuits in which there is extensive basal inhibition, not captured by the model because of the zero basal firing rate, may be poorly simulated in our model. Further, circuits with extensive neuromodulation or extrasynaptic signalling will be poorly modelled. This is consistent with recent studies showing that activity propagation measured by calcium imaging is not well predicted by anatomical connectivity alone in *Caenorhabditis elegans*, demonstrating the importance of extrasynaptic signalling not accounted for in our model^50^. Finally, precise dynamics may be poorly simulated by our computational model, although a similar LIF model accurately produced *Drosophila* ring attractor dynamics^18^.

## *Drosophila* taste coding

Our computational and experimental results generate new contributions to our understanding of *Drosophila* taste coding. First, we identify that sugar and water form a shared attractive pathway. We have found previously that hunger impinges primarily at sugar-sensing neurons, and at select second-order neurons; water-sensing neurons also are tuned by thirst^4^. By altering the tuning of water- or sugar-sensing neurons at the periphery, followed by funnelling their activity onto a shared appetitive circuit, the *Drosophila* brain may allow for a compact representation of attractive tastants. We also find that Ir94e is an aversive taste modality; Ir94e GRNs in virgin females have been shown to respond to the presentation of male genitals^38^. Additionally, we find that bitter and Ir94e inhibit proboscis extension by impinging on pre-MNs.

A strength of computational modelling in general is that it is explicit about its assumptions and limitations. In this simple leaky integrate-and-fire model, we treat each neuron identically as a spiking neuron and ignore neural morphology as well as different neurotransmitter receptor dynamics^17^. Furthermore, the model does not account for gap junctions, non-spiking neurons, internal state or long-range neuropeptides, and assumes that the basal firing of each neuron is zero^51–55^. In addition, the accuracy of the model is limited by the underlying synapse and neurotransmitter prediction accuracy^3,21^. Moreover, studies of the connectomes of *C.* *elegans* and the crustacean stomatogastric ganglion demonstrate that connectivity knowledge constrains, but does not dictate, a particular circuit mechanism^56–58^. Despite these limitations, the model performs remarkably well for the demonstrated use cases. Across 164 predictions we were able to test empirically, 91% were consistent with our empirical results (Supplementary Table 9). Excluding our optogenetic split-GAL4 experiments (Fig. 2), in which the vast majority of cell types did not elicit MN9 activation, the accuracy of the model is 84% (Supplementary Table 10). Further refinements of our computational model, for example, more complete neurotransmitter or receptor information, or more sophisticated treatment of the morphology of each neuron, may improve the accuracy of future models. We show here that, in the intermediate complexity of the entire *Drosophila* brain, a simple connectome-based computational model can reliably describe entire sensorimotor transformations from sensory input to descending or motor output.

## Methods

### Computational model

We implement a leaky integrate-and-fire model as previously described^18,19,59–61^ with α-synapse dynamics, using the following three differential equations and parameters:

$${\rm{d}}{v}_{i}/{\rm{d}}t=({g}_{i}-({v}_{i}-{V}_{{\rm{resting}}}))/{T}_{{\rm{mbr}}}$$

$${\rm{d}}{g}_{i}/{\rm{d}}t=-{g}_{i}/\tau $$

$${g}_{i}\leftarrow {g}_{i}+{w}_{j,i}:{\rm{u}}{\rm{p}}{\rm{o}}{\rm{n}}\,{\rm{s}}{\rm{p}}{\rm{i}}{\rm{k}}{\rm{e}}\,{\rm{f}}{\rm{r}}{\rm{o}}{\rm{m}}\,{\rm{n}}{\rm{e}}{\rm{u}}{\rm{r}}{\rm{o}}{\rm{n}}\,j$$

*V*_resting_ = −52 mV (resting potential from ref. ^18^);

*V*_reset_ = −52 mV (reset potential after spike^18^);

*V*_threshold_ = −45 mV (threshold for spiking^18^);

*R*_mbr_ = 10  Kohm cm^2^ (membrane resistance^18^);

*T*_refractory_ = 2.2 ms (refractory period^18,59^);

*C*_mbr_ = 2 µF cm^−2^ (membrane capacitance^18^);

*T*_mbr_ = *C*_mb_ × *R*_mbr_ (definition of membrane timescale in a resistor–capacitor circuit);

*τ* = 5 ms (synapse decay timescale^61^);

*T*_dly_ = 1.8 ms (time delay from spike to change in membrane potential from ref. ^62^);

*W*_syn_ = 0.275 mV (free parameter; synaptic weight, that is, how much each synapse influences downstream membrane potential);

*g*_*i*_ (the synaptic conductance resulting from the aggregate firing of neurons presynaptic to neuron *i*).

In this model, *V*_*i*_, the membrane potential of neuron *i*, decays back to *V*_resting_, the resting potential, in the absence of any stimulus. If an upstream neuron, *j*, fires, the membrane potential changes in proportion to the connectivity (*w*_*j*,*i*_). If the upstream neuron is excitatory, the neuron depolarizes; if inhibitory, the neuron hyperpolarizes.

All parameters are taken from previous *Drosophila* modelling or electrophysiology efforts^18,19,61,62^, from the synaptic weights from the Flywire connectome (public materialization v.630)^18,19^, or from the neurotransmitter predictions^3,63^, except for *W*_syn_, the single free parameter of the model, which corresponds to how much the downstream membrane potential changes as a result of a single excitatory or inhibitory synapse. We chose *W*_syn_ such that activation of sugar GRNs at 100 Hz resulted in roughly 80% of maximal MN9 firing^64,65^.

The connection weight, *w*_*j*,*i*_, between neuron *j* and neuron *i* is the synaptic connectivity weight from the Flywire connectivity multiplied by either 1, if neuron *j* is excitatory or −1, if neuron *j* is inhibitory, multiplied by *W*_syn_.

We used α-synapse modelling as performed previously^17,59^ (https://brian2.readthedocs.io/en/stable/user/converting_from_integrated_form.html). Upon firing of the upstream neuron, the conductance variable *g*_*i*_ is revised: *g*_*i*_ becomes *g*_*i*_ + *w*_*j*,*i*_. *g*_*i*_, upon initialization of the network, or after firing of the neuron, starts at 0 mV. Because the membrane potential dynamics are defined by:

$${\rm{d}}{v}_{i}/{\rm{d}}t=({g}_{i}-({v}_{i}-{V}_{{\rm{resting}}}))/{T}_{{\rm{mbr}}}$$

a change in *g*_*i*_ changes the potential that the neuron will now decay towards.

Furthermore, *g*_*i*_ exponentially decays with the timescale of *τ*:

$${\rm{d}}{g}_{i}/{\rm{d}}t=-{g}_{i}/\tau $$

Therefore, after an initial change in membrane potential, once *g*_*i*_ decays back towards 0, the membrane potential again decays back to the resting potential. Upon firing, a neuron’s membrane potential is reset to the resting potential, and cannot change for the duration of the refractory time period.

We stimulated particular neurons with Poisson distributed input. The neural model is implemented in Brian2 (ref. ^22^), and 30 simulations of 1,000 ms for each experiment were performed. Simulation of sugar neuron activation takes approximately 5 min per 1,000 ms trial per central processing unit thread. All 127,400 proofread neurons from Flywire materialization v.630 are included in the model.

### Neurotransmitter predictions

Neurotransmitter predictions are from ref. ^3^. In this dataset, the neurotransmitter are predicted for each synapse. We assume GABAergic and glutamatergic neurons are inhibitory^66^, and that each neuron is either exclusively inhibitory or excitatory. As in ref. ^67^, we used a cleft score cutoff of 50, and identified the highest neurotransmitter prediction for each presynaptic site and, if greater than half of all the presynaptic sites across the entire neuron are predicted to be inhibitory (GABA or Glut), we assigned this neuron as inhibitory. Neurons predicted to be dopaminergic, octopaminergic or serotonergic are assigned to the excitatory category. In the entire Flywire volume, approximately 55% of neurons are predicted to be cholinergic, 24% glutamatergic, 14% GABAergic and the remaining 7% are predicted to be dopaminergic, octopaminergic or serotonergic^3^. Among the 613 taste responsive that respond when sugar/water are activated to elicit 40 Hz MN9 firing, or Ir94e/bitter are activated to eliminate 40 Hz MN9 firing (Supplementary Table 4), the neurotransmitter breakdowns are as follows: 52% are predicted to use acetylcholine, 25.9% GABA, 17% glutamate, 2.9% serotonin, 2.0% dopamine and 0.2% octopamine.

### Assessment of model robustness to parameters and assumptions

To determine how robust our model is to *W*_syn_, the weight parameter, we varied *W*_syn_ by increasing or decreasing *W*_syn_ by 30% (Supplementary Table 11). We compared the prediction of the model in each of the three parameters (decreased by 30%, normal, increased) across the 164 different computational experiments for which we have experimental data. We modulated the sensory input to compensate for this change (that is, increased sugar firing when *W*_syn_ was decreased). In the condition in which we decreased *W*_syn_ by 30%, the decreased *W*_syn_ model predictions were consistent with 90.2% of the default model predictions; the decreased accuracy of the *W*_syn_ model was 85%—a decrease of 91% from the accuracy of the default model. A notable exception is that decreasing *W*_syn_ by 30% results in a significant decrease in the ability of water GRNs to activate MN9. The model in which *W*_syn_ was increased by 30% had predictions that were consistent with the default model across 95% of the 164 predictions; these predictions were 88% accurate. Therefore, we conclude that our model is robust to perturbations to the free parameter *W*_syn_. Because scaling *W*_syn_ essentially results in scaling the ‘distance’ between the resting potential and the firing threshold potential, we did not test the robustness of the model to changes to the resting potential or firing threshold. Additionally, because changes to the membrane time constant, as well as *T*_dly_ (the time delay from spike to change in membrane potential) and the refractory period (*T*_refractory_) generally simply change overall firing rates by permitting more (or less) firing, we did not subject these to robustness checks as we already vary the input firing rates (and have tested increasing *W*_syn_, which fundamentally alters firing rates).

Consistent with previous *Drosophila* computational modelling efforts, we assume that excitatory and inhibitory synapses have the same magnitude^18^. To test how robust the model is to this assumption, we varied the inhibitory:excitatory weight ratio by 50% (Supplementary Table 11). Decreasing the strength of inhibition results in predictions consistent with 95% of those of the default model; accuracy drops from 91% to 88%. The increased synapse strength model makes predictions consistent with the default model 96% of the predictions, with an accuracy of 89%. In total, we conclude that the computational model is robust across changes to *W*_syn_ and the inhibitory:excitatory synaptic strength ratio.

Although glutamate can be either excitatory or inhibitory in *Drosophila*, by default we assume that glutamate is inhibitory, consistent with previous experimental work and computational modelling^1,18,66,68,69^. At present, it is not feasible to predict whether a particular glutamatergic connection is excitatory or inhibitory at a brainwide scale. To test the consequences of our assumption that glutamate is inhibitory, we instead assumed glutamate is excitatory and examined how this change altered the predictions of the model. Although many of the model’s predictions were consistent between these two assumptions (Supplementary Table 11f), we found two main differences. First, the computational result that bitter and Ir94e are inhibitory is eliminated if we assume glutamate is excitatory. Further, altering this assumption increases the false positive rate of the optogenetic activation experiment (Fig. 2) from 1% to 16%.

### Computational modelling limitations

We model neurons as either excitatory or inhibitory spiking neurons, which generally models GABAergic and cholinergic neurons reasonably well, but other neurons (for example, dopaminergic or serotonergic) will be modelled less well. We assume each neuron is either exclusively inhibitory or excitatory. We ignore neural morphology and receptor dynamics. The underlying synapses or neurotransmitter predictions may not be fully accurate. Gap junctions cannot be identified in the electron microscopy dataset, so we ignore their possibility. We do not account for neuropeptides or neuromodulation. Furthermore, we assume a basal firing rate of 0 Hz. An important consequence of this assumption is that inhibitory connections to an inactive neuron have no effect. Because of these assumptions, the absolute firing rates of the model are unlikely to be accurate. Rather, we prefer to interpret broad differences in firing rates between different conditions (for example, increasing bitter stimulation results in decreasing MN9 firing), across a range of input firing rates.

### Computational modelling of water and sugar GRN activity

GRNs were activated at particular frequencies using Poisson distributed input to generate 10–200 Hz firing (sugar) or 20–260 Hz firing (water). Because GRN reconstructions on the left hemisphere are more complete^23^, probably due to challenges imaging and reconstructing neurons at the brain’s periphery, we performed unilateral left hemisphere activation for all simulations, except for Extended Data Fig. 1d. The firing times of all neurons that fired in any of 30 simulations was recorded, and then these data were converted into average firing per second. The top 200 firing neurons were then activated at 25–200 Hz, and the firing of all neurons was recorded. For computational silencing, water or sugar GRNs were activated, and each of the top 200 firing neurons was silenced by eliminating all output of that neuron. Unless specifically stated, when describing MN9 activity, we refer to the MN9 contralateral to the GRNs activated.

### Classification of GRNs

Bitter and Ir94e GRNs were identified previously by their distinctive morphology^23^. Water and sugar GRNs were identified previously by morphology; to confirm these assignments and to incorporate new GRNs identified in Flywire, we performed hierarchical clustering using the connectivity of GRNs onto second-order neurons, using cosine distance as a measure of similarity^70^. This identified three broad clusters of GRNs (Extended Data Fig. 1a). We compared this clustering with the response properties of second-order neurons in starved flies, the nutritional state for which we have the most complete data^4^. Cluster 2 GRNs synapse onto, among others, G2N-1 and FMIN, which respond exclusively to sugar. Cluster 3 neurons synapse comparatively strongly onto Fudog and Phantom, which respond to both water and sugar in starved flies; cluster 3 neurons also synapse more strongly, on average, onto Usnea than cluster 2 neurons. Usnea responds exclusively to water. Thus, we conclude that cluster 2 corresponds to sugar GRNs, consistent with their previous assignment^23^, and cluster 3 corresponds to water GRNs. We speculate that cluster 1 GRNs are Ppk23-glut GRNs^31^ that may respond to salt; however, little is known about this pathway, making testing this hypothesis challenging. We also examined the connectivity of ‘zero-order’ gustatory neurons onto cluster 2 and 3 GRNs. This clustering corresponds strongly with the GRN to second-order clustering (Extended Data Fig. 1), verifying that these clusters reflect distinct taste modalities. As a conservative measure, we chose to examine only those sugar GRNs that fully correspond between the GRN to second-order clustering and the zero-order to GRN clustering.

Recently, the FlyWire brain (FAFB) dataset (on the basis of which the Flywire dataset was prepared) was found to be left–right inverted^1,2^. In this paper, we refer to the true biological side. Thus, we note that ‘right hemisphere GRNs’ in this paper correspond to ‘left hemisphere GRNs’ described previously^4,23^.

### Identification of SEZ split-GAL4 neurons in the Flywire volume and computational activation

A total of 106 out of 138 cell types of SEZ split-GAL4 library were identified in the Flywire volume^44,71^. Two semi-independent methods were used to identify these neurons: in the first method, aligned JRC2018 unisex registered brains were downloaded from https://splitgal4.janelia.org and, where possible, single neuron multicolour Flp-out imagery was used. Neurons were skeletonized in FIJI by selecting a threshold that eliminated the background, but retained the morphology of the neuron. Neurons were skeletonized using the ‘Skeletonize 2D/3D’ tool, and files were saved as a .nrrd file. These .nrrd files were converted to .swc in natverse^72^, and uploaded to the Flywire gateway (https://flywiregateway.pniapps.org/upload). This generated pointclouds that were used to manually identify the neuron of interest.

In the second method, we identified the SEZ interneurons in Flywire with the aid of NBLAST^73^. The morphology of SEZ split-GAL4 interneurons from single neuron multicolour Flp-out images were provided in the dotprops format^44^, from which the pointcloud of each neuron was extracted. The pointcloud was transformed from the JRC2018U brain to FAFB using the Navis and FlyBrains libraries. To narrow down the candidate neurons in FlyWire near the pointcloud, we assigned between one and four three-dimensional boxes surrounding the dense regions of the pointcloud. The neuronal segments in the boxes were queried using the CloudVolume input/output interface of FlyWire. The skeletons of neuronal segments were calculated and their similarities to the dotprops of the SEZ interneuron were measured using NBLAST. The candidates with the highest NBLAST scores were compared visually with the pointcloud in the three-dimensional view of FlyWire for final decision.

SEZ split-GAL4 neurons were identified independently between the two methods and sets of researchers, and only those for which there was a clear consensus were used. All identified neurons in a cell type were activated computationally, and the resulting MN9 firing rate was recorded.

### Modelling of the antennal grooming circuit

A total of 147 JONs belonging to classes JO-C, JO-E, JO-F and JO-mz were identified previously and described in the electron microscopy volume^7^. As in the feeding initiation circuit, JONs were activated to generate 20–220 Hz firing. The firing times of all neurons that fired in any of 30 1,000 ms simulations were recorded, and these data were then converted into average firing per second. The top 300 firing neurons were then activated at 50, 100, 150 and 200 Hz to determine whether they could activate aDN1 or aDN2. All 147 JONs were activated, each of the 300 top firing neurons was silenced by eliminating all outputs of that neuron and the activity of aDN1 or aDN2 was recorded. aBN1, aBN2, aDN1 and aDN2 were each identified and annotated from their previously described morphology^2,6,7^.

### Chrimson optogenetic activation experiments

PER was scored as previously described^4,74^. Female flies were raised on standard cornmeal-yeast-molasses medium. At 48 h before experiments, flies were placed on molasses food with 0.4 mM retinal. Flies (3–5 days old) were anesthetized with carbon dioxide, mounted onto a glass slide with nail polish and allowed to recover for 2 h in a humidified chamber at 22 °C. For optogenetic activation experiments, 153 μW mm^−2^ 635 nm laser light was used (Laserglow). Flies were scored for whether they extended their proboscis within a 5 s period in response to light. Experiments were performed blind to genotype. For the screen, ten flies per genotype were scored for any movement of the proboscis. Split-GAL4s with any extension were scored a second time from a second, independent, cross specifically for extension of the rostrum, that is, MN9 activation. If several split-GAL4 lines were tested, the split-GAL4 line with the highest MN9 is shown in Fig. 2b.

### Silencing experiments

Three-day-old female flies were raised on standard cornmeal-yeast-molasses medium, and transferred to standard food with 0.4 mM all-trans retinal for 48 h. Flies were anesthetized with carbon dioxide, mounted onto a glass slide with nail polish and desiccated for 3 h in a sealed chamber with around 250 g CaSO_4_ (Drierite, catalogue no. 23001) at 22 °C (ref. ^75^). A green laser (532 nm, LaserGlow LBS-532) was used to acutely silence neurons using GtACR1 (ref. ^76^). Flies presented with water three times to the proboscis, and the number of flies that extended at least once was recorded. For Kir2.1 and *Amontillado* RNAi experiments, experiments were performed as above, except that no all-trans retinal or green laser was used.

### Ir94e and Gr66a optogenetic activation

Experiments were performed as in the GtAcr1 experiments, except that flies were exposed to red light rather than green light^77^. Flies were raised on 0.4 mM retinal in standard food for 4 days. Flies were anesthetized with carbon dioxide, mounted onto a glass slide with nail polish and allowed to recover for 2 h in a humidified chamber at 22 °C. 153 μW mm^−2^ 635 nm laser light was used (Laserglow). Flies were water satiated, then presented with either 50 mM sucrose or 1 M sucrose three times to the proboscis, and the number of flies that extended at least once was recorded. Experiments were performed blind to genotype.

### Calcium imaging setup for Fudog and Zorro imaging

Mated female flies were dissected for calcium imaging studies 14–21 days posteclosion as previously described^4,29^. Flies were anesthetized briefly with ice as they were placed in a custom plastic holder at the cervix to isolate the head from the rest of the body. The head was then immobilized using ultraviolet light activated glue, and the oesophagus was cut to provide unobstructed imaging access to the SEZ. For Fudog, flies were food-deprived in a vial containing a wet kimwipe for 18–24 h before imaging. Following dissection, samples were bathed in artificial hemolymph-like solution (AHL) (around 250 mOsmo) and imaged immediately. To generate thirsty-like (pseudodesiccated) Zorro flies^4^, following dissection, samples were bathed in high AHL (around 350 mOsmo) and allowed to rest for 1 h before imaging.

### Calcium imaging of Fudog and Zorro

For imaging responses to taste solutions, female flies of UAS-CD8-tdTomato;20XUAS-IVS-GCaMP6s(attP5);20XUAS-IVS-GCaMP6s(VK00005) were crossed to male flies for each split-GAL4 line, and female progeny without balancers were selected for imaging. The following tastants were used: double-distilled water (‘water’), 1 M sucrose (‘sugar’) or 10 mM denatonium plus 100 mM caffeine in 20% polyethylene glycol (‘bitter’). Taste solutions were delivered to the proboscis using a glass capillary (1.0 mm outer diameter and 0.78 mm inner diameter) filled with approximately 4 µl of taste solution and positioned at the tip of the proboscis using a micromanipulator. Taste solutions were drawn away from the tip of the capillary at the beginning of each imaging trial using slight suction generated by an attached 1 ml syringe, and delivered to the proboscis at the relevant time during imaging with light pressure applied to the syringe.

We performed one-photon imaging of Fudog as described previously^4^ using a 3i spinning disc confocal microscope with a piezo drive and a ×20 water immersion objective (numerical aperture = 1.0) with a ×2.5 magnification changer. A total of 55 frames of eight *z* sections spaced at 1 µM intervals were binned in lots of four by four and acquired at 0.8 Hz using a 488 nm laser. Taste solutions were in contact with the proboscis labellum from frame 20 to frame 25.

We performed two-photon imaging of Zorro as described previously^4^ using a Scientifica Hyperscope with resonant scanning, a piezo drive and a ×20 water immersion objective (numerical aperture = 1.0) with a ×4 digital zoom. A total of 80 stacks of 20 *z* sections spaced at 2 µM intervals were acquired at 0.667 Hz using a 920 nm laser. Taste solutions were in contact with the proboscis labellum from frame 30 to frame 40.

### Functional connectivity between JON subpopulations and aBN1

JON functional connectivity experiments were performed as described previously^8^. This experiment required the use of two binary expression systems (GAL4/UAS and LexA/LexAop) for driving the expression of CsChrimson and GCaMP6 in different neurons in the same fly. CsChrimson was expressed in the JON subpopulations using LexA and spGAL4 driver lines that were specific for either JO-CE or -F neurons. Regions of interest specific to aBN1 were chosen, and CsChrimson was excited by delivering 2 ms pulses of 590 nm light through the objective through a light-emitting diode.

### Taste response calcium imaging analysis

For calcium imaging analysis of Extended Data Figs. 1c and  3a, image analysis was carried out in Fiji^78^, CircuitCatcher (a customized Python program by D. Bushey), Python, and R. First, in Fiji, *z* stacks for each timepoint were maximum intensity projected and then movement corrected using the StackReg plugin with ‘Rigid Body’ or ‘Translation’ transformation^79^. Next, using CircuitCatcher, a region of interest (ROI) containing the neurites of the cell type of interest was selected along with a background ROI, and average fluorescence intensity for each ROI at each timepoint was retrieved. Then, in Python, background subtraction was carried out for each timepoint (*F*_t_). To calculate *F*_initial_, initial fluorescence intensity was calculated as the mean corrected average fluorescence intensity from frames 9–18 (for one-photon imaging) or frames 0–19 (for two-photon imaging and optogenetic imaging). Finally, the following formula was used to calculate Δ*F*/*F* = *F*_t_ − *F*_initial_/*F*_initial_. Area under the curve was approximated with the trapezoidal rule in Python using the NumPy.trapz function. Area under the curve was assessed from frames 20 to 25 (for one-photon imaging).

### Statistical analysis

Analysis of behavioural assays and computational modelling were performed in Prism. Fisher’s exact test was used to compare the fraction of PER responses in experimental versus control flies. Two-tailed Mann–Whitney *U* test was used for comparison of computational modelling predictions. Statistical analysis of calcium imaging was carried out in R, Python (taste imaging) and Julia (JON functional connectivity). We performed Quade’s test with Quade’s All Pairs test using Holm’s correction for the taste-stimulation calcium imaging experiments. All statistical tests used were two-sided, except for the Wilcoxon one-sided test to test whether tastant calcium imaging responses were greater than zero. All behavioural data shown were replicated independently successfully at least twice; for behavioural experiments, one replicate is one fly. Raw data for behavioural data can be found in Supplementary Table 9.

### Reporting Summary

Further information on research design is available in the Nature Portfolio Reporting Summary linked to this article.

## Online content

Any methods, additional references, Nature Portfolio reporting summaries, source data, extended data, supplementary information, acknowledgements, peer review information; details of author contributions and competing interests; and statements of data and code availability are available at 10.1038/s41586-024-07763-9.

## Supplementary information

Reporting Summary

Supplementary Tables Supplementary Tables 1–12.

## Data Availability

Results from the computational modelling described in the paper can be found at https://edmond.mpdl.mpg.de/dataset.xhtml?persistentId=doi:10.17617/3.CZODIW. Behavioural data can be found in Supplementary Table 9.

## Code availability

The brain model is available at https://github.com/philshiu/Drosophila_brain_model.
