# Supplementary material for: A Drosophila computational brain model reveals sensorimotor processing
Source: Nature. 2024 Oct 2;634(8032):210–9. doi: 10.1038/s41586-024-07763-9 (PMC11446845; doi:10.1038/s41586-024-07763-9)
Supplement: Supplementary file 1 — Reporting Summary [file 41586_2024_7763_MOESM1_ESM.pdf]

nature portfolio

Corresponding author(s): Philip Shiu, Kristin Scott

Last updated by author(s): January 8, 2024

Reporting Summary

Nature Portfolio wishes to improve the reproducibility of the work that we publish. This form provides structure for consistency and transparency in reporting. For further information on Nature Portfolio policies, see our Editorial Policies and the Editorial Policy Checklist.

Statistics

For all statistical analyses, confirm that the following items are present in the figure legend, table legend, main text, or Methods section.

n/a	Confirmed	
☐	☒	The exact sample size (*n*) for each experimental group/condition, given as a discrete number and unit of measurement
☐	☒	A statement on whether measurements were taken from distinct samples or whether the same sample was measured repeatedly
☐	☒	The statistical test(s) used AND whether they are one- or two-sided<br>*Only common tests should be described solely by name; describe more complex techniques in the Methods section.*
☐	☒	A description of all covariates tested
☐	☒	A description of any assumptions or corrections, such as tests of normality and adjustment for multiple comparisons
☐	☒	A full description of the statistical parameters including central tendency (e.g. means) or other basic estimates (e.g. regression coefficient) AND variation (e.g. standard deviation) or associated estimates of uncertainty (e.g. confidence intervals)
☐	☒	For null hypothesis testing, the test statistic (e.g. *F*, *t*, *r*) with confidence intervals, effect sizes, degrees of freedom and *P* value noted<br>*Give P values as exact values whenever suitable.*
☒	☐	For Bayesian analysis, information on the choice of priors and Markov chain Monte Carlo settings
☒	☐	For hierarchical and complex designs, identification of the appropriate level for tests and full reporting of outcomes
☒	☐	Estimates of effect sizes (e.g. Cohen's *d*, Pearson's *r*), indicating how they were calculated

Our web collection on statistics for biologists contains articles on many of the points above.

Software and code

Policy information about availability of computer code

Data collection	2-photon microscopy images were collected using ScanImage (Vidrio Technologies, version 2019). Light pulses for optogenetic activation were controlled by custom Matlab code. The custom computational model was written in Python (version 3.10.12) and the Brian2 library (version 2.5.4). Creation of the model also used the Python libraries fafbseg version 1.15 and CaveClient 5.4.2. The computational model is publicly available at https://github.com/philshiu/Drosophila_brain_model/tree/main. Identification of neurons of interest used the Navis Python library, Natverse R library (1.8.22), and the online Flywire Gateway tool (https://flywiregateway.pniapps.org/)
Data analysis	Statistical analysis of behavioral data was performed in Prism (Version 10.0.0) and the Python package Scipy (version 1.10.1). Image analysis was performed in Fiji (version 2.9.0/ImageJ 1.53t). Analysis of taste calcium imaging timeseries was performed using CircuitCatcher (a customized Python program by Daniel Bushey Dag et al., 2019), followed by custom Python code.<br>Statistical analysis of taste calcium imaging was carried out in both R and Python. For taste imaging experiments carried out in a block design with three treatments, Quade and Quade all pairs test with holm p-adjust were carried out in R using the PMCMRplus package (version 1.9.7). Quade test was chosen because it is more powerful than Friedman for a block-design experiment with three treatments (Conover, 1999).<br>One-sample Wilcoxon signed rank tests of taste calcium imaging were carried out in Python using the SciPy Python package(version 1.7.3).

For manuscripts utilizing custom algorithms or software that are central to the research but not yet described in published literature, software must be made available to editors and reviewers. We strongly encourage code deposition in a community repository (e.g. GitHub). See the Nature Portfolio guidelines for submitting code & software for further information.

Data

Policy information about availability of data

All manuscripts must include a data availability statement. This statement should provide the following information, where applicable:

- Accession codes, unique identifiers, or web links for publicly available datasets
- A description of any restrictions on data availability
- For clinical datasets or third party data, please ensure that the statement adheres to our policy

All data from this paper is available: Results from the computational modeling described in the paper can be found at https://edmond.mpdl.mpg.de/dataset.xhtml?persistentId=doi:10.17617/3.CZODIW
Behavioural data can be found in supplemental table 9.

Research involving human participants, their data, or biological material

Policy information about studies with human participants or human data. See also policy information about sex, gender (identity/presentation), and sexual orientation and race, ethnicity and racism.

Reporting on sex and gender	*Use the terms sex (biological attribute) and gender (shaped by social and cultural circumstances) carefully in order to avoid confusing both terms. Indicate if findings apply to only one sex or gender; describe whether sex and gender were considered in study design; whether sex and/or gender was determined based on self-reporting or assigned and methods used. Provide in the source data disaggregated sex and gender data, where this information has been collected, and if consent has been obtained for sharing of individual-level data; provide overall numbers in this Reporting Summary. Please state if this information has not been collected. Report sex- and gender-based analyses where performed, justify reasons for lack of sex- and gender-based analysis.*
Reporting on race, ethnicity, or other socially relevant groupings	*Please specify the socially constructed or socially relevant categorization variable(s) used in your manuscript and explain why they were used. Please note that such variables should not be used as proxies for other socially constructed/relevant variables (for example, race or ethnicity should not be used as a proxy for socioeconomic status). Provide clear definitions of the relevant terms used, how they were provided (by the participants/respondents, the researchers, or third parties), and the method(s) used to classify people into the different categories (e.g. self-report, census or administrative data, social media data, etc.) Please provide details about how you controlled for confounding variables in your analyses.*
Population characteristics	*Describe the covariate-relevant population characteristics of the human research participants (e.g. age, genotypic information, past and current diagnosis and treatment categories). If you filled out the behavioural & social sciences study design questions and have nothing to add here, write "See above."*
Recruitment	*Describe how participants were recruited. Outline any potential self-selection bias or other biases that may be present and how these are likely to impact results.*
Ethics oversight	*Identify the organization(s) that approved the study protocol.*

Note that full information on the approval of the study protocol must also be provided in the manuscript.

Field-specific reporting

Please select the one below that is the best fit for your research. If you are not sure, read the appropriate sections before making your selection.

☒ Life sciences ☐ Behavioural & social sciences ☐ Ecological, evolutionary & environmental sciences

For a reference copy of the document with all sections, see nature.com/documents/nr-reporting-summary-flat.pdf

Life sciences study design

All studies must disclose on these points even when the disclosure is negative.

Sample size	We used standard values from the field, and similar to our previous papers (e.g., Shiu et al., 2022 eLife).
Data exclusions	No data was excluded.
Replication	All behavioral experiments were reproduced independently at least twice.
Randomization	Control and experimental flies were scored in random order during behavioral experiments.
Blinding	Behavioral experiments were performed with the experimenter blinded to genotype.

Reporting for specific materials, systems and methods

We require information from authors about some types of materials, experimental systems and methods used in many studies. Here, indicate whether each material, system or method listed is relevant to your study. If you are not sure if a list item applies to your research, read the appropriate section before selecting a response.

Materials & experimental systems

n/a	Involved in the study
☒	☐ Antibodies
☒	☐ Eukaryotic cell lines
☒	☐ Palaeontology and archaeology
☐	☒ Animals and other organisms
☒	☐ Clinical data
☒	☐ Dual use research of concern
☒	☐ Plants

Methods

n/a	Involved in the study
☒	☐ ChIP-seq
☒	☐ Flow cytometry
☒	☐ MRI-based neuroimaging

Animals and other research organisms

Policy information about studies involving animals; ARRIVE guidelines recommended for reporting animal research, and Sex and Gender in Research

Laboratory animals

Three- to five-day-old Drosophila melanogaster flies were scored for behavioral experiments. 14–21 days post-eclosion mated female flies were used for the tastant calcium imaging. For imaging responses to taste solutions, females of UAS-CD8-tdTomato;20XUAS-IVS-GCaMP6s(attP5);20XUAS-IVS-GCaMP6s(VK00005) were crossed to males for each split-GAL4 line, and female progeny without balancers were selected for imaging. Genotypes of split-Gal4 lines are described in Sterne et al., 2021. The following strains were used: Drosophila: 20XUAS-IVS-CsChrimson.mVenus}attP18
Drosophila: UAS-GtACR1.d.EYFP}attP2
Drosophila: Zorro split-GAL4, SS67405
Drosophila: Clavicle split-GAL4, SS48947
Drosophila: G2N-1 split-GAL4, SS47082
Drosophila: Rattle split-GAL4, SS50091
Drosophila: Usnea split-Gal4, SS37122
Drosophila: Phantom split-GAL4, SS44877
Drosophila: Fudog split-GAL4, SS35290
Drosophila: Bract split-GAL4, SS31320
Drosophila: Tophat split-GAL4, SS39932
Drosophila: Tulip split-GAL4, SS44899
Drosophila: Roundup split-GAL4, SS47745
Drosophila: w[1118];
Drosophila: UAS-Kir2.1 (II)
Drosophila: UAS-Amontillado RNAi;
20XUAS-IVS-GCaMP6s(attP40);
Drosophila: Gr64f-Gal4 (II)
Drosophila melanogaster: UAS-CD8-tdTomato;;
Drosophila melanogaster: w[1118]; 20XUAS-IVS-GCaMP6s(attP40);
Drosophila melanogaster: w[1118];; 20XUAS-IVSGCaMP6s(VK00005)
Drosophila: 20XUAS-IVS-jGCaMP7b(attP5)
Drosophila melanogaster: IR94e-Gal4
Drosophila: 20XUAS-IVS-jGCaMP7b(VK00005)

Wild animals

No wild animals were used in these experiments.

Reporting on sex

All proboscis extension and taste calcium imaging experiments were performed on female flies, consistent with previous experiments (Shiu et al., 2022), and the electron microscopy volume is based on a female brain.

Field-collected samples

No samples were collected from the field.

Ethics oversight

No ethics approval or oversight is necessary for Drosophila experiments.

Note that full information on the approval of the study protocol must also be provided in the manuscript.
